# DNA Mismatch Repair Protein Immunohistochemistry and MLH1 Promotor Methylation Testing for Practical Molecular Classification and the Prediction of Prognosis in Endometrial Cancer

**DOI:** 10.3390/cancers10090279

**Published:** 2018-08-21

**Authors:** Jisup Kim, Jin Kyoung Kong, Wookyeom Yang, Hanbyoul Cho, Doo Byung Chay, Bang Hyun Lee, Seong Jin Cho, Soonwon Hong, Jae-Hoon Kim

**Affiliations:** 1 Department of Pathology, Yonsei University College of Medicine, 146-92 Dogok-Dong, Gangnam-Gu, Seoul 06273, Korea; jspath@yuhs.ac (J.K.); soonwonh@yuhs.ac (S.H.); 2 Department of Obstetrics and Gynecology, Gangnam Severance Hospital, Yonsei University College of Medicine, 146-92 Dogok-Dong, Gangnam-Gu, Seoul 06273, Korea; haidami@yuhs.ac (J.K.K.); wookyeom@yuhs.ac (W.Y.); hanbyoul@yuhs.ac (H.C.); chaydb@yuhs.ac (D.B.C.); 3 Department of Obstetrics and Gynecology, Kangdong Sacred Heart Hospital, Hallym University College of Medicine, 18, Cheonho-daero 173-gil, Gangdong-gu, Seoul 05355, Korea; Banghyun.lee@gmail.com; 4 Department of Pathology, Kangdong Sacred Heart Hospital, Hallym University College of Medicine, 18, Cheonho-daero 173-gil, Gangdong-gu, Seoul 05355, Korea; apilas@hanmail.net

**Keywords:** endometrial cancer, molecular classification, mismatch repair deficiency, MLH1 promoter methylation test

## Abstract

The incidence of endometrial cancer is rapidly increasing worldwide, and its molecular classification has gained importance for new therapeutic approaches. This study sought to examine the clinicopathologic features and immune markers associated with the DNA mismatch repair (MMR) status and MLH1 promoter methylation status of endometrial cancer patients. A total of 173 patients with primary endometrial cancer who had received a hysterectomy were evaluated for four MMR proteins (MLH1, MSH2, MSH6, and PMS2), immune markers (CD8, programmed cell death protein 1 (PD-1), and programmed death-ligand 1 (PD-L1)) and p53 by immunohistochemistry (IHC), followed by an MLH1 methylation test. Patients were classified into MMR deficiency or proficiency, sporadic cancer, or probable Lynch syndrome (PLS), and the clinicopathologic features (including the expression of peritumoral immune markers) and prognosis of each group were compared. Patients with MMR deficiency or PLS showed an increase in immune markers compared those with MMR proficiency or sporadic cancer, respectively, and PLS demonstrated higher immune marker expression than MLH1 promoter methylation. Regarding prognosis, patients with MMR deficiency showed significant adverse overall survival (OS) when in stages I and II. Practical molecular classifications based on p53 staining results, in addition to MMR or PLS status, revealed an increased predictive ability for OS compared with the European Society of Medical Oncologists (ESMO) risk groups. The results of this study suggest that PLS may be a better candidate for an immune checkpoint inhibitor than MMR deficiency. The practical molecular classification contributes not only to the screening of Lynch syndrome, but also assists in predicting the prognosis in endometrial cancer.

## 1. Introduction

Endometrial cancer is the most common gynecologic malignancy in the developed world; its incidence is rapidly increasing, and carries the highest disease burden [1,2].

Approximately 2–5% of endometrial cancer cases are presumed to be caused by genetic factors, of which Lynch syndrome is the most common [3]. Lynch syndrome is an autosomal dominant genetic condition caused by germline mutations in DNA mismatch repair (MMR) genes, leading to microsatellite instability (MSI) [3]. Universal screening is recommended for all newly diagnosed endometrial cancer patients, both for the purpose of the prevention of secondary cancer as well as for the prevention of cancer development in close family members [4]. Suggested universal screening includes not only immunohistochemistry (IHC) for the four MMR proteins (MLH1, MSH2, MSH6, PMS2), but also MLH1 methylation testing for patients with a loss of MLH1/PMS2 expression; approximately 30% of MMR protein loss is caused by MLH1 promoter methylation, resulting in epigenetic silencing [4,5].

Endometrial cancer has been traditionally classified into two major subtypes: estrogen-related type I cancer, which is predominantly of an endometrioid histotype with favorable prognosis; and estrogen-unrelated type II cancer, which is a predominantly non-endometrioid histotype with unfavorable prognosis [6]. This classification has been challenged due to the clinicopathologic, biologic, and genetic heterogeneity of tumors, as well as for its inability to guide treatment modality [7]. The Cancer Genome Atlas (TCGA) yielded four molecular subgroups of endometrial cancer (polymerase E catalytic subunit (POLE) ultra-mutated, MSI hyper-mutated, copy number (CN) low, and CN high), based on nucleotide substitution patterns and frequencies, MSI status, and copy number cluster [8]. More cost-effective and practical classification has been developed based on TCGA data by the ProMisE/Vancouver group [9] and the Leiden/TransPORTEC group [9,10]. The molecular classifications revealed different prognoses among the subgroups, with the worst prognosis demonstrated in the p53 mutation subgroup [8,9,10] and the best prognosis in the POLE-mutated group; however, contradictory reports do exist [11,12].

The POLE- and MSI-mutated subgroups harbor high neoantigen loads, increased immune checkpoint expression (such as programmed cell death protein 1 (PD-1) and programmed death-ligand 1 (PD-L1)), as well as increased numbers of tumor-infiltrating lymphocytes (TILs); these findings suggest that these molecular subgroups are good candidates for immunotherapy [13,14]. Additionally, there are ongoing clinical trials for MSI endometrial cancer [15,16].

Testing for a POLE mutation may be not yet be pragmatic in daily practice, as there is only limited evidence to guide therapeutic options. Furthermore, the cost-effectiveness of the test is an issue, in that testing of the hotspot (P286R and V411L) mutation covers only two-thirds of the POLE mutation [17]. There is also controversy surrounding the prognostic value of the POLE mutation, unlike TCGA data [11,12]. On the contrary, MLH1 promoter methylation is included in the universal Lynch screening protocol, which is recommended for patients diagnosed with endometrial cancer [4,5].

This study aimed to classify endometrial cancer patients according to their MLH1 promoter methylation status instead of the POLE mutation test, as well as by their p53 and MMR status, and to correlate these classifications with clinicopathologic features, including prognosis and immune marker expression. Such a classification scheme may provide valuable insight for more refined, personalized therapy (reflecting heterogeneous pathogenesis) and more refined risk stratification in endometrial cancer.

## 2. Results

### 2.1. Clinicopathologic Characteristics

**Figure 1:**
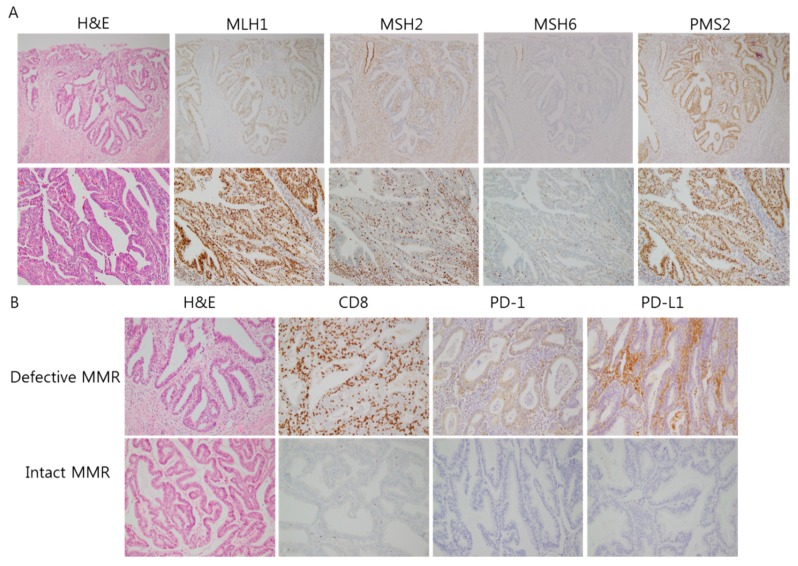
(**A**) Immunohistochemistry of four mismatch repair (MMR) proteins, showing the expression of MLH1/PMS2 and loss of MSH2/MSH6 (upper: ×100, lower: ×200). (**B**) Immunohistochemistry of CD8, PD-1, and PD-L1, showing differential peritumoral expression between defective and intact MMR proteins (×200).

The baseline clinical and pathologic characteristics of the study population are represented in Table 1, and the IHC for the MMR proteins are illustrated in Figure 1A. In the IHC analysis and MLH1 methylation test for the primary endometrioid carcinoma from 173 patients, loss of MMR IHC was observed in 45 cases (26.0%). Cases with a loss of MLH1/PMS2, MSH2/MSH6, MSH6, and isolated PMS2 loss totaled 26 (15.0%), 12 (6.9%), 5 (2.8%), and 2 (1.1%) cases, respectively. Among patients with MLH1/PMS2 loss, 8 cases (30.8%) revealed an unmethylated MLH1 promoter, and 18 cases (69.2%) revealed a methylated MLH1 promoter. Accordingly, 27 cases (15.6%) were regarded as PLS, and the remaining 146 cases (84.4%) were regarded as sporadic cancer.

MMR deficiency was associated with postmenopausal status, tumor grades 2–3, FIGO stages I–II (*p* = 0.010, 0.011, 0.006), BMI < 25, and parous state (parity ≥ 1) with borderline significance (*p* = 0.051, 0.064). PLS was associated with BMI < 25, FIGO stage I–II, (*p* = 0.015, 0.034) and with parous state with borderline significance (*p* = 0.052) (Table 2). Among the patients with sporadic cancer, MLH1 methylated cases were associated with tumor grade 3 (*p* = 0.019), presence of lymphovascular invasion (*p* = 0.023), and a larger tumor size (51.7 ± 26.3 mm versus 35.1 ± 22.4 mm) compared to patients with MMR proficiency.

### 2.2. Peritumoral Immune Marker Expression

**Figure 2:**
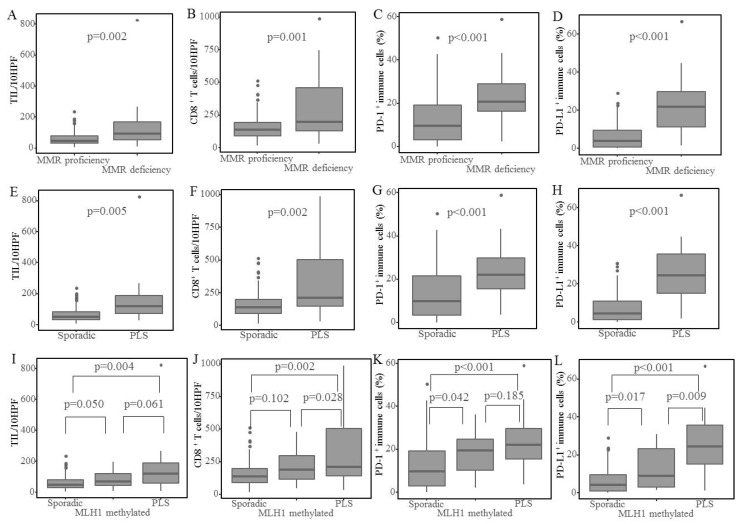
Peritumoral immune marker expression. (**A**–**D**) Immune marker expression in MMR proficient vs MMR deficient tumors, including (**A**) TIL, (**B**) CD8^+^ T lymphocytes, (**C**) peritumoral PD-1^+^ immune cells, and (**D**) peritumoral PD-L1^+^ immune cells. (**E**,**F**) Immune marker expression in sporadic cancer vs PLS cases, including (**E**) TIL, (**F**) CD8^+^ T lymphocytes, (**G**) peritumoral PD-1^+^ immune cells, and (**H**) peritumoral PD-L1^+^ immune cells. (**I**–**K**) Immune marker expression in sporadic vs MLH1 methylated vs PLS cases, including (**I**) TIL, (**J**) CD8^+^ T lymphocytes, (**K**) peritumoral PD-1^+^ immune cells, and (**L**) peritumoral PD-L1^+^ immune cells. The horizontal line in each box shows the median, and the top and bottom boundary lines of the box show the 75th and 25th percentiles, respectively. The vertical lines above and below the boxes show the maximum and minimum observations within 1.5 IQR (interquartile range) of the upper and lower quartile. The points beyond the vertical line are outliers beyond the 1.5 IQR of the upper and lower quartile.

Peritumoral immune marker expression is illustrated in Figure 1B. The MMR-deficient group showed higher numbers of TILs and peritumoral CD8^+^ T lymphocytes than the MMR-proficient group (*p* = 0.002, 0.001), and the PLS group showed higher numbers than the sporadic group (*p* = 0.005, 0.002). Regarding immunoreactivity to PD-1 and PD-L1 in peritumoral immune cells, the MMR-deficient group showed a higher immunoreactivity than the MMR-proficient group (*p* < 0.001). The PLS group showed higher immunoreactivity than the sporadic cancer group (*p* < 0.001), although PD-L1 expression in tumor cells revealed no significant difference (*p* = 0.256, 0.155) (Table 3 and Figure 2).

Among the cases with sporadic cancer, MLH1 methylated cases showed higher numbers of peritumoral PD-1^+^ immune cells (*p* = 0.042) and peritumoral PD-L1^+^ immune cells (*p* = 0.017), and a tendency for higher numbers of peritumoral CD8^+^ T lymphocytes (*p* = 0.102), compared to MMR-proficient cases (Figure 2).

Among the MMR-deficient cases, the PLS group revealed significantly higher numbers of CD8^+^ T lymphocytes (*p* = 0.028) and peritumoral PD-L1^+^ immune cells (*p* = 0.009), and a tendency for higher numbers of peritumoral PD-1^+^ immune cells (*p* = 0.185) and PD-L1^+^ tumor cells (*p* = 0.093), compared to sporadic cancer with MLH1 methylation cases (Figure 2).

### 2.3. Prognosis

**Figure 3:**
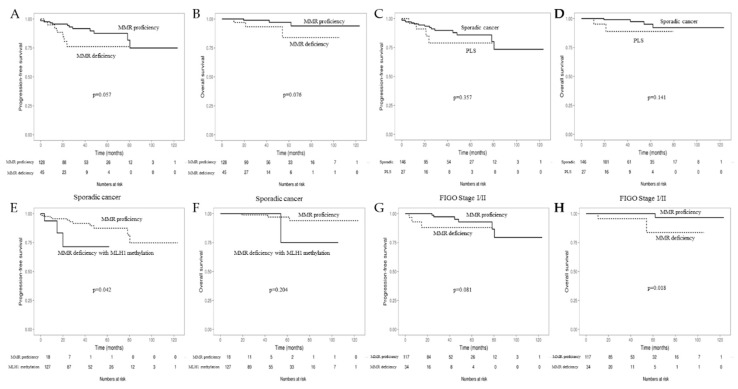
Kaplan–Meier curves for progression-free survival (PFS) and overall survival (OS) according to the DNA mismatch repair (MMR) immunohistochemistry (IHC) status and MLH1 promotor methylation status for endometrial cancer. PFS (**A**) and OS (**B**) in the MMR-deficient vs the MMR-proficient group. PFS (**C**) and OS (**D**) in the sporadic cancer vs the PLS group. Comparison of PFS (**E**) and OS (**F**) in the sporadic cancer group between those with MMR deficiency with MLH1 methylation and those with MMR proficiency. PFS (**G**) and OS (**H**) of the MMR-deficient vs the MMR proficient group in the patients with FIGO stages I–II.

Cases with MMR deficiency showed a trend of poor progression-free survival (PFS) and overall survival (OS) (*p* = 0.057, 0.076) (Figure 3A,B). Although PLS was not associated with PFS (*p* = 0.357), it showed a trend of poor OS (*p* = 0.141) (Figure 3C,D). Among the sporadic cancer cases, MMR deficiency with a methylated MLH1 promoter showed poor PFS compared to MMR-proficient cases (*p* = 0.042); however, no significant difference in OS was observed between the two groups (*p* = 0.204) (Figure 3E,F). Among patients with FIGO stages I–II, MMR deficiency showed a trend for poor PFS (*p* = 0.081) and a significantly poorer prognostic value for OS (*p* = 0.018) (Figure 3G,H).

**Figure 4:**
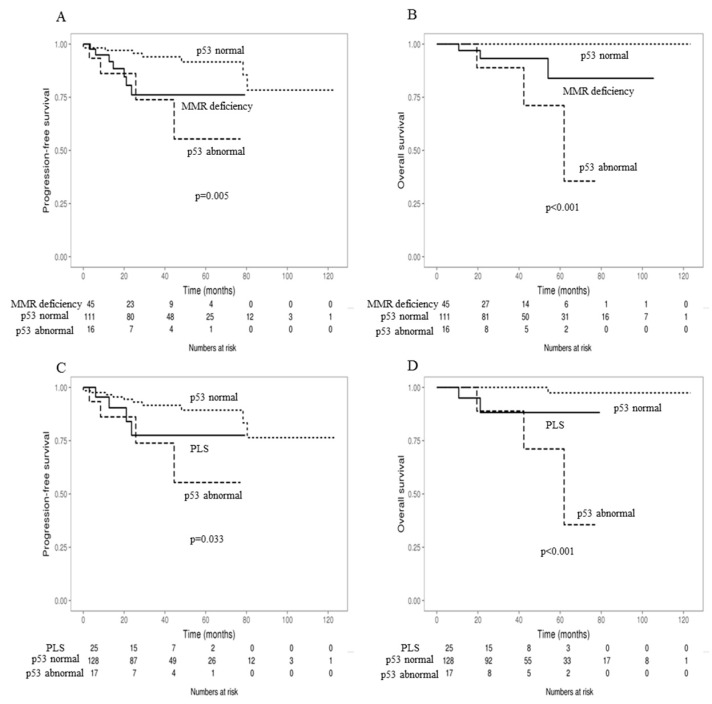
Kaplan–Meier curves for progression-free survival (PFS) (**A**) and overall survival (OS) (**B**), according to the practical molecular classification of endometrial cancer, based on DNA mismatch repair (MMR) status and p53 staining results in endometrial cancer patients. Kaplan–Meier curves for progression-free survival (PFS) (**C**) and overall survival (OS) (**D**), according to the practical molecular classification of endometrial cancer based on probable Lynch syndrome (PLS) status and p53 staining results in endometrial cancer patients.

Practical molecular classification of endometrial cancer according to the MMR status and p53 staining results (MMR 3-tier classification), revealed the best PFS and OS in MMR proficiency with normal p53, followed by MMR deficiency, and finally, MMR proficiency with abnormal p53 (*p* = 0.005, <0.001) (Figure 4A,B).

Practical molecular classification of endometrial cancer, according to PLS and p53 staining results (PLS 3-tier classification) revealed that the best PFS and OS in sporadic cancer with wild type p53, followed by PLS, and finally, sporadic cancer with mutation type p53 (*p* = 0.033, <0.001) (Figure 4C,D).

**Figure 5:**
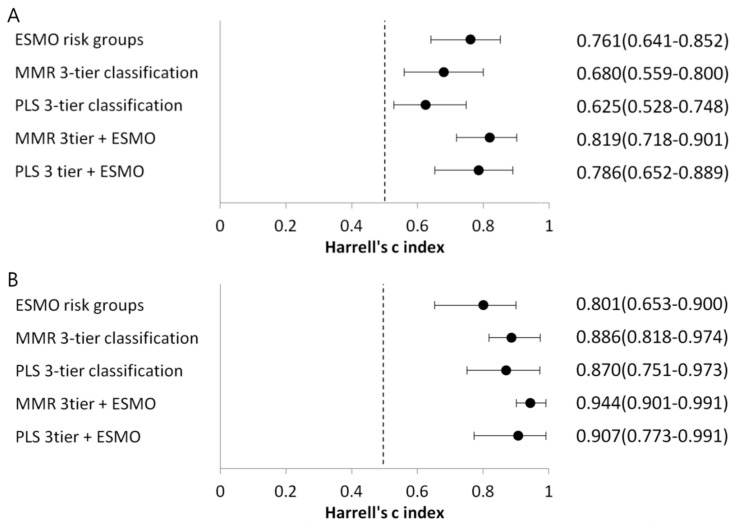
Harrell’s C-index for progression-free survival (PFS) (**A**) and overall survival (OS) (**B**). The combination of European Society of Medical Oncologists (ESMO) risk groups with MMR three-tier classification or PLS three-tier classification improved the predictive ability for both PFS and OS. For OS, MMR three-tier classification or PLS three-tier classification demonstrated a better predictive ability than the ESMO risk groups.

The predictive ability of the molecular classifications for PFS and OS was evaluated by Harrell’s C-index [18]. For comparison, clinical risk groups were assigned to three groups, according to the European Society of Medical Oncologists (ESMO) risk groups—group 1: low to intermediate (118 cases), group 2: high-intermediate (11 cases), group 3: high to advanced/metastatic (44 cases) (Supplementary Table S1) [19]. Molecular classifications were also combined with ESMO risk groups. The C-statistics of ESMO risk groups, MMR three-tier classification, PLS three-tier classification, MMR three-tier classification with ESMO risk groups, and MMR three-tier classification with ESMO risk groups were 0.761, 0.680, 0.625, 0.819, and 0.786, respectively, for PFS and 0.801, 0.886, 0.870, 0.944, and 0.907 for OS, respectively (Figure 5). MMR three-tier classification and PLS three-tier classification demonstrated better predictive ability than the ESMO risk groups for OS, although not for PFS. Also, the predictive ability improved for PFS and OS when ESMO risk groups were combined with MMR three-tier classification or PLS three-tier classification (Figure 5).

## 3. Discussion

According to previous literature regarding unselected endometrial cancer patients, the frequency of MMR deficiency ranges from 20 to 40%, and PLS from 8 to 13% [20,21,22]. In this study, MMR deficiency was found in 26.0% (45/173) of cases and PLS in 15.6% (27/173) of cases, which is consistent with the existing results.

Regarding clinicopathologic features, MMR deficiency was associated with postmenopausal status, a higher histologic grade (G2–3), and a lower FIGO stage (I–II). In a previous study, MMR deficiency was associated with younger age (<60), parity (≥1), and a lower BMI (<30) [20]; these results are similar to the present study, in that MMR deficiency was associated with parity and lower BMI, with borderline significance for the two, although there was no association between age and MMR status. In the present study, PLS was associated with lower BMI (<25) and a lower FIGO stage (I–II), both of which were consistent with a previous study [20].

An adverse prognostic tendency of MMR deficiency was demonstrated for PFS and OS, as was a significantly poorer OS in the FIGO stage I–II subgroup. There have been several conflicting reports on the prognosis of MMR deficiency (or MSI); some reports revealed a favorable prognosis of MMR deficiency, while others revealed an adverse prognosis of MMR deficiency [20,23,24,25]. Indeed, several reports have revealed no association of MMR deficiency with prognosis [26,27]. The present study also revealed an adverse prognostic tendency of PLS for OS, which is in contrast to a previous study that revealed a favorable prognosis of PLS [20]. Another report revealed no difference in prognosis between the PLS (MSI/MLH1 unmethylated), MSI/MLH1 methylated, and microsatellite stable (MSS) groups [26].

It was interesting to find that MMR deficiency and PLS showed a tendency for adverse prognosis, in spite of their association with a low FIGO stage as well as higher TIL and peritumoral CD8^+^ T lymphocytes, which are predictors of favorable prognosis. However, the tendency of poor prognosis of MMR deficiency and PLS could be understood in that these conditions possess an increased surface expression of immune checkpoint inhibitors. There are contradictory reports on the prognostic value of MMR deficiency, and reports on the prognosis of PLS remain relatively rare. Consequently, subsequent studies are required for validation of the prognostic values.

The present study also revealed adverse PFS of methylated MLH1 promoter cases, compared to intact MMR cases, among patients with sporadic cancer. The prognostic difference may be explained by the association of MLH1 promotor methylation cases with a high histologic grade, lymphovascular invasion, larger tumor size, and a higher expression of PD-1 and PD-L1 in peritumoral immune cells. A previous study reported significantly reduced recurrence-free survival and larger tumor volume with the MMR-deficient with MLH1 methylation group compared to the MMR proficient group, which is in agreement with results from the present study [28]. MLH1 promotor methylation has also been associated with adverse prognosis in cancer of other organs, such as the ovary and colon [29,30]. The prognostic significance in this study may suggest that MMR deficiency caused by somatic mutation (epigenetic silencing of MLH1 promotor) is an important cause of genomic instability, such as germline mutation in Lynch syndrome, which subsequently leads to aggressive behavior of the tumor.

The POLE mutated group is regarded as having favorable prognosis and the p53 mutated group is regarded as having poor prognosis [8,9,10]; although contradictory studies exist regarding the POLE mutation [11,12]. Although TCGA study revealed no prognostic difference between MSI (hypermutated) group and copy number low (endometrioid) group, the ProMisE study (Vancouver) and the PORTEC study (Leiden), revealed poor prognosis of the MMR-deficient or MSI group compared to the p53 wild-type or no specific molecular profile (NSMP) group [8,9,10].

The incidence of POLE mutation in endometrial cancer patients is known to be 7–12% [8,14]. POLE mutations are common in patients with stage I, grade 3 cancers, who usually require adjuvant therapy [10]. In this patient group, the POLE mutation test may help to avoid unnecessary adjuvant therapy, given that the POLE mutation is a reliable predictor of a good prognosis. However, more evidence is required in order to make a decision to withhold adjuvant therapy based on a POLE mutation [17]. In addition, given the high cost of the POLE mutation test, and the absence of certainty with regards to whether or not to exclude adjuvant therapy, questions may arise as to whether the test would be effective.

Although molecular classification of endometrial cancer utilizing POLE mutation testing has been developed and widely studied in the TCGA, Vancouver, and Leiden classifications, such molecular analysis still has limitations, namely in that POLE mutation testing is too expensive to be carried out in daily routine practice. Furthermore, the MMR-deficient group includes sporadic cancer, in which MMR deficiency occurs by MLH1 promoter methylation, as well as the PLS group where it occurs by germline mutation of DNA MMR genes [20]; the above-mentioned molecular classifications do not distinguish these heterogeneous groups [8,9,10].

Given the necessity to devise methods that can be more broadly applied to patients, the present study aims to make a practical molecular classification of endometrial cancer as three subgroups, using MMR and p53 immunohistochemistry (MMR 3-tier classification). MMR loss cases were classified as the MMR defective group, and MMR-proficient patients were classified according to the p53 immunohistochemistry result. The worst prognosis was demonstrated in the p53 abnormal subgroup, and the best prognosis was seen in the p53 normal (NSMP) subgroup; these results are similar to those of the Vancouver and Leiden classifications, with the exception that the POLE mutation group was excluded from the present study.

Endometrial cancer was further classified into three subgroups using the MLH1 methylation test, as well as MMR and p53 immunohistochemistry (PLS three-tier classification). PLS patients were classified first, and the remaining sporadic cancer patients were classified according to their p53 immunohistochemistry result. The p53 abnormal subgroup revealed the worst prognosis, and the p53 wild type, among sporadic cancer, revealed the best prognosis; finally, PLS revealed an intermediate prognosis.

As mentioned previously, abnormal p53 showed the worst prognosis in both of the classifications in the present study; this may be explained by the aggressive oncogenic effect as well as a loss of tumor suppressor function, leading to malignant transformations such as tumor initiation, promotion, aggressiveness, and metastasis [31]. In this study, there were no significant differences in immunological profiling between the p53 abnormal and normal subgroups in either classification (Supplementary Material Figures S1 and S2), which is in line with a previous study based on the Leiden/PORTEC classification [14].

The practical molecular classifications (MMR three-tier classification and PLS three-tier classification) were directly compared with the ESMO risk groups, which are the strongest guide for direct adjuvant therapy [19]. Both practical molecular classifications revealed a higher Harrell’s C-index than the ESMO risk groups for the prediction of OS, although not for PFS. Also, a combination of the molecular classifications with ESMO risk groups revealed an increased Harrell’s C-index, compared with ESMO risk groups alone. These results suggest that molecular classifications are useful risk stratification systems.

Regarding the immune profile, the present study revealed that MMR loss and PLS were associated with high TIL, CD8^+^ T lymphocytes, PD-1^+^ peritumoral immune cells, and PD-L1^+^ peritumoral immune cells. In a previous study by Pakish et al. [32], MSI-high cases were associated with higher Granzyme B^+^ cells (representing activated cytotoxic T lymphocyte (CTL), or natural killer cells), PD-L1^+^ cells, CD3^+^ cells, CD4^+^ cells, and CD8^+^ Granzyme B^+^ cells (activated CTL) in the tumor stroma, compared with MSS cases; PLS cases were associated with higher CD8^+^ cells (CTL), CD8^+^ Granzyme B^+^ cells (activated CTL), and PD-L1^+^ CD68^+^ cells (PD-L1^+^ macrophages). These results were, for the most part, consistent with the current study. Howitt et al. [13] also reveal an overexpression of PD-1 and PD-L1 in peritumoral lymphocytes, and higher numbers of CD3^+^ and CD8^+^ TIL, as well as a greater neoantigen load in POLE and MSI tumors compared to MSS tumors.

PD-L1, expressed on the surface of various immune cells, and to a greater extent on some tumor cells, binds to its ligand, PD-1, to form an inhibitory signal; the resulting negative regulation plays a key role in the suppression of alloimmune responses, protecting against host damage from overactive T-cells by inducing immune tolerance. However, in the context of cancer, overexpression of PD-L1 by cancer cells facilitates evasion of the anti-tumor immune response by inhibiting T cell-mediated tumor cell killing [33,34,35]. The monoclonal antibody blocking this pathway has shown a durable response in melanoma, non-small cell lung cancer, renal cell carcinoma, and bladder cancer [36]. PD-L1 has been reported to be expressed in the majority of primary and metastatic endometrial cancers [37], and there are ongoing clinical trials utilizing PD-L1 antibodies in advanced endometrial cancer [38]. Another clinical trial revealed an improved response to anti-PD-1 therapy in MMR-deficient tumors, including endometrial cancer; this was based on the hypothesis that hypermutated tumors have increased expression of tumor-specific antigens [36].

In this study, the MMR loss and PLS group revealed not only a higher number of TILs and peritumoral CD8^+^ T lymphocytes, but also a greater immunoreactivity to PD-1 and PD-L1 in peritumoral immune cells. In addition, the comparison of MMR deficiency cases showed that the PLS group had increased peritumoral immune marker expression (such as CD8^+^, PD-L1^+^, and PD-1^+^) compared to the MLH1 methylation cases. Accordingly, targeting the PD-1/PD-L1 pathway in MMR-deficient or PLS patients may predict an excellent response and prognosis. Since it has been previously reported that MMR deficiency could be a good candidate for immunotherapy, the current study sought to focus on the PLS group as a better candidate for immunotherapy [38].

The study has a number of limitations. First, POLE mutation testing, utilized for molecular classification of endometrial cancer, was not performed; however, POLE testing may not be cost-effective for daily routine practice. Further studies including POLE mutation testing would help to validate the usefulness of the practical molecular classifications suggested in the study. Second, the retrospective cohort study included a single cohort with a relatively short follow-up duration. Third, the number of cases in each group of the suggested classifications is not the same, and warrants further research employing a similar number of patients in each group for a more precise comparison. Fourth, multivariable analysis was not performed. However, a similar prognostic tendency was acquired with previous molecular classification, utilizing MMR immunohistochemistry, a MLH1 methylation test, and p53 immunohistochemistry, which are utilized currently for universal Lynch screening protocol in daily practice. Such classifications may provide insight for a more refined, personalized therapy reflecting heterogeneous pathogenesis, and for a more refined risk stratification in endometrial cancer.

## 4. Materials and Methods

### 4.1. Patients and Specimens

This retrospective cohort study included 173 consecutive endometrial cancer patients who were diagnosed with endometrioid histology following a hysterectomy at Gangnam Severance Hospital and Gangdong Sungsim Hospital from 2006 to 2016, and who had adequate tumor tissue for analysis. All cancer samples were obtained from the Korean Gynecologic Cancer Bank. Medical records of the patients were reviewed, and the median follow up duration was 27 months (range of 1–123 months). Although none of the patients received neoadjuvant therapy, 50 patients underwent postoperative adjuvant therapy according to their stage and grade, including radiotherapy, chemotherapy, or concurrent chemoradiotherapy. The study protocol was approved by the Institutional Review Board of Gangnam Severance Hospital on 17 October 2016 (ethics code: 3-2016-0236), and informed consent was waived.

Cases showing intact MMR expression or loss of MLH1/PMS2 with MLH1 promoter methylation were regarded as sporadic cancer, and cases showing a loss of MSH2/MSH6, isolated loss of MSH6 or PMS2, and a loss of MLH1/PMS2 without MLH1 promoter methylation were regarded as probable Lynch syndrome (PLS) [20].

### 4.2. Immunohistochemistry

The review of hematoxylin and eosin slides were performed by two experienced pathologists (J.S.K. and S.W.H.), and one representative block was selected for each case. Immunohistochemistry was performed on the representative paraffin-embedded 4 μm-thick whole tissue sections using a BenchMark XT automated stainer (Ventana Medical Systems, Tucson, AZ, USA) for CD8 (clone CD/144B, 1:100; Cell Marque, Rocklin, CA, USA), p53 (clone DO-7, 1:3000; Novocastra, Buffalo Grove, IL, USA) and MMR proteins, and a Leica automated stainer (Bond Max) for PD-1 (clone NAT105, 1:100; Cell marque) and PD-L1 (clone E1L3N, 1:150; Cell Signaling, Danvers, MA, USA). Antibodies used for MMR proteins were anti-human MLH1 (clone M1, ready to use; Ventana, Oro Valley, AZ, USA), MSH2 (clone G219-1129, ready to use; Ventana), MSH6 (clone 44, ready to use; Ventana), and PMS2 (clone ERP3947, ready to use; Ventana) monoclonal antibodies. Immunohistochemical evaluation was performed by two experienced pathologists (J.S.K. and S.W.H.) blinded to clinical data. Loss of MMR proteins (MMR deficiency) was defined as a complete loss of nuclear staining in the tumor cells with positive internal controls (stromal cells, lymphocytes, or normal endometrium). CD8 expression was evaluated as the number of CD8^+^ T lymphocytes in 10 randomly selected high-power fields (HPFs). P53 staining was scored as 0 (completely negative), 1 (focal, weak to moderate positive), and 2 (diffuse > 80%, strong positive); scores of 0 and 2 were regarded as abnormal, and a score of 1 was regarded as normal [39]. PD-1 and PD-L1 expression in immune cells were evaluated as the percentage of PD-1^+^ or PD-L1^+^ immune cells relative to the total number of immune cells in tumor stroma. PD-L1 expression in tumor cells was evaluated as the percentage of PD-L1^+^ tumor cells showing membranous expression relative to total number of tumor cells.

### 4.3. MLH1 Promoter Methylation Test

The MLH1 promoter methylation test was performed in patients with MLH1/PMS2 loss. For MLH1 promoter methylation analysis, sodium bisulfite modification of genomic DNA (2 μg) was performed using Epitect Bisulfite Kits (Qiagen, Hilden, Germany) following DNA extraction from formalin-fixed paraffin-embedded tissue with QIAamp DNA FFPE Tissue Kit (Qiagen). After dissolving in 20 μL of distilled water, methylated DNA and unmethylated control DNA (Qiagen) were added to bisulfite-treated DNA (100 mg). Bisulfite-treated DNA served as a template for methylation-specific polymerase chain reactions (PCR), using primers specific to methylated and unmethylated CpG islands of the MLH1 promoter. The forward and reverse primers for the methylated MLH1 promoter were 5′-GATAGCGATTTTTAACGC-3′ and 5′-TCTATAAATTACTAAATCTCTTCG-3′, respectively. The forward and reverse primers for the unmethylated MLH1 promoter were 5′-AGAGTGGATAGTGATTTTTAATGT-3′ and 5′-ACTCTATAAATTACTAAATCTCTTCA-3′, respectively. Amplification was performed using a PTC-200 thermocycler (MJ Research Inc., Waltham, MA, USA), and the amplified PCR products were electrophoresed on 2.5% agarose gel containing ethidium bromide to confirm the methylation status (Supplementary Figure S3).

### 4.4. Statistical Analysis

For descriptive statistics, continuous variables were expressed as mean ± standard deviation (SD), and categorical variables were expressed as number and percentage. A Student *t*-test and Chi-square test (or Fisher’s exact test) was performed for continuous and categorical variables, respectively. Kaplan–Meier survival curves were plotted, and a logrank test was used to compare progression-free survival (PFS) and overall survival (OS). To investigate the predictive ability of the practical molecular classifications, Harrell’s C-index was calculated [18].

All analyses were conducted using SPSS version 23.0 (IBM, Armonk, NY, USA) and R software, version 3.2.5 for Windows (the R foundation for statistical computing, Vienna, Austria), and *p*-values < 0.05 were considered statistically significant; *p*-values between 0.05 and 0.15 were considered as a trend toward significance.

## 5. Conclusions

In conclusion, sub-classification of the MMR-deficient group revealed higher immune checkpoint expression, as well as increased immune cell infiltration in PLS patients compared to those with sporadic cancer with MLH1 promotor methylation. These results suggest that the PLS group may be a better candidate for immune checkpoint inhibitors. Furthermore, practical molecular classification of endometrial cancer utilizing MMR IHC or MLH1 promotor methylation test ± p53 IHC would not only help in screening Lynch syndrome, but also to predict the prognosis of endometrial cancer patients; this in turn would aid in determining the extent of surgery and adjuvant therapeutic modality, including targeted therapy.

## Figures and Tables

**Table 1:** Baseline clinical and pathologic characteristics of the study population.

Patient Characteristics	Number	%
Age (mean ± SD)	54.0 ± 9.8	
BMI (kg/m^2^) (mean ± SD)	25.1 ± 4.9	
Menopausal status		
Pre-menopause	74	43.0
Post-menopause	98	57.0
Parity status		
Nulliparous	31	18.0
Parous	141	82.0
FIGO Stage		
I	139	80.3
II	12	6.9
III	17	9.8
IV	5	2.9
Histology		
Endometrioid	173	100
Tumor grade		
1	89	51.7
2	55	32.0
3	28	16.3
Myometrial invasion		
<1/2	117	68.4
≥1/2	54	31.6
Lymphovascular invasion		
Absent	139	82.7
Present	29	17.3
Lymph node metastasis		
Absent	159	93.0
Present	12	7.0
Tumor size		
≤2 cm	35	27.6
>2 cm	92	72.4
Postoperative adjuvant therapy		
Not performed	123	71.1
Performed	50	28.9
P53 staining		
Normal (IHC 1+)	149	87.6
Abnormal (IHC 0 or 2+)	21	12.4

**Table 2:** Comparison of clinicopathologic characteristics, according to DNA mismatch repair (MMR) status and probable Lynch syndrome (PLS) (versus sporadic cancer).

Clinicopathologic Variables	MMR Status			PLS or Sporadic Cancer		
	Proficiency	Deficiency	*p*-Value *	Sporadic	PLS	*p*-Value *
	*n* = 128	*n* = 45		*n* = 146	*n* = 27	
Age			0.258			0.974
<50	49(38.3)	13(28.9)		53(36.3)	9(33.3)	
≥50	79(61.7)	32(71.1)		93(63.7)	18(66.7)	
BMI			0.051			0.015
<25	64(52.0)	31(68.9)		74(52.5)	21(77.8)	
≥25	59(48.0)	14(31.1)		67(47.5)	6(22.2)	
Menopausal status			0.010			0.051
Pre-menopause	62(48.8)	12(26.7)		67(46.2)	7(25.9)	
Post-menopause	65(51.2)	33(73.3)		78(53.8)	20(74.1)	
Parity status			0.064			0.052
Nulliparous	27(21.3)	4(8.9)		30(20.7)	1(3.7)	
Parous	100(78.7)	41(91.1)		115(79.3)	26(96.3)	
FIGO Stage			0.014			0.028
I	108(84.4)	31(68.9)		121(82.9)	18(66.7)	
II	9(7.0)	3(6.7)		10(6.8)	2(7.4)	
III	8(6.3)	9(20.0)		12(8.2)	5(18.5)	
IV	3(2.3)	2(4.4)		3(2.1)	2(7.4)	
Tumor grade			0.011			0.213
1	73(57.5)	16(35.6)		78(53.8)	11(40.7)	
2–3	54(42.5)	29(64.4)		67(46.2)	16(59.3)	
Myometrial invasion			0.157			0.264
<1/2	90(71.4)	27(60.0)		101(70.1)	16(59.3)	
≥1/2	36(28.6)	18(40.0)		43(29.9)	11(40.7)	
Lymphovascular invasion			0.003			0.055
Absent	109(87.9)	30(68.2)		121(85.2)	18(69.2)	
Present	15(12.1)	14(31.8)		21(14.8)	8(30.8)	
Lymph node metastasis			0.083			0.407
Absent	120(95.2)	39(86.7)		135(93.8)	24(88.9)	
Present	6(4.8)	6(13.3)		9(6.3)	3(11.1)	
Tumor size			0.539			0.242
≤2 cm	27(29.0)	8(23.5)		28(25.5)	7(41.2)	
>2 cm	66(71.0)	26(76.5)		82(74.5)	10(58.8)	
Postoperative adjuvant therapy			0.007			0.016
Not performed	98(76.6)	25(5.6)		109(74.7)	14(51.9)	
Performed	30(23.4)	20(44.4)		37(25.3)	13(48.1)	
P53 staining			0.867			0.743
Normal (IHC 1+)	111(87.4)	38(88.4)		128(88.3)	21(84.0)	
Abnormal (IHC 0 or 2+)	16(12.6)	5(11.6)		17(11.7)	4(16.0)	

* A Chi-square test (or Fisher’s exact test) was used to test the association between two categorical variables.

**Table 3:** Comparison of the tumor-infiltrating lymphocytes (TIL) count, CD8^+^ T lymphocyte count, and programmed cell death protein 1 (PD-1) and programmed death-ligand 1 (PD-L1) expression, according to DNA mismatch repair (MMR) status and probable Lynch syndrome (PLS) (versus sporadic cancer).

Variable	MMR Status		
	Proficiency Mean *±* SD (*n* = 128)	Deficiency Mean *±* SD (*n* = 45)	*p*-Value
Tumor-infiltrating lymphocytes/10HPFs	59.62 ± 44.55	125.52 ± 127	0.002
Peritumoral CD8^+^ T lymphocytes/10FPFs	157.55 ± 101.68	295.69 ± 221.73	0.001
Peritumoral PD-1^+^ immune cells (%)	12.17 ± 11.39	22.22 ± 12.23	<0.001
Peritumoral PD-L1^+^ immune cells (%)	6.21 ± 6.85	21.89 ± 14.44	<0.001
Tumor cell PD-L1^+^ cells (%)	1.19 ± 4.81	2.79 ± 7.69	0.256
**Variable**	**PLS or Sporadic Cancer**		
	**Sporadic Mean *±* SD (*n* = 146)**	**PLS Mean *±* SD (*n* = 27)**	***p*-Value**
Tumor-infiltrating lymphocytes/10HPFs	62.55 ± 46.49	155.27 ± 152.98	0.005
Peritumoral CD8^+^ T lymphocytes/10FPFs	163.9 ± 105.44	352.14 ± 252.75	0.002
Peritumoral PD-1^+^ immune cells (%)	13.02 ± 11.49	24.48 ± 12.82	<0.001
Peritumoral PD-L1^+^ immune cells (%)	7.24 ± 7.92	26.93 ± 14.47	<0.001
Tumor cell PD-L1^+^ cells (%)	1.11 ± 4.51	4.29 ± 9.7	0.155

## Supplementary Materials

The following are available online at http://www.mdpi.com/2072-6694/10/9/279/s1, Figure S1: Peritumoral immune marker expression according to the practical molecular classification of endometrial cancer based on DNA mismatch repair (MMR) status and p53 mutation status in endometrial cancer patients. (A) TIL, (B) CD8^+^ T lymphocytes, (C) peritumoral PD-1^+^ immune cells, and (D) peritumoral PD-L1^+^ immune cells; Figure S2: Peritumoral immune marker expression according to the practical molecular classification of endometrial cancer based on probable Lynch syndrome (PLS) status and p53 mutation status in endometrial cancer patients. (A) TIL, (B) CD8^+^ T lymphocytes, (C) peritumoral PD-1^+^ immune cells, and (D) peritumoral PD-L1^+^ immune cells; Figure S3: Results of MLH1 promotor methylation test showing amplified un methylation and methylation PCR products, A: Unmethylated human control DNA MLH1 methylation PCR; B: Methylated human control DNA (bisulfite converted); C: Unmethylated human control DNA (bisulfite converted); 1: Not significant result due to material insufficiency; 2: A case showing MLH1 methylation; 3: A case showing no MLH1 methylation; Table S1: European Society of Medical Oncologists (ESMO) risk groups.
