# Supplementary material for: DNA Mismatch Repair Protein Immunohistochemistry and MLH1 Promotor Methylation Testing for Practical Molecular Classification and the Prediction of Prognosis in Endometrial Cancer
Source: Cancers (Basel). 2018 Aug 21;10(9):279. doi: 10.3390/cancers10090279 (PMC6162750; doi:10.3390/cancers10090279)
Supplement: Supplementary file 1 [file cancers-10-00279-s001.pdf]

Supplementary Materials: DNA Mismatch Repair Protein Immunohistochemistry and MLH1 Promotor Methylation Testing for Practical Molecular Classification and the Prediction of Prognosis in Endometrial Cancer

Jisup Kim, Jin Kyoung Kong, Wookyeom Yan, Hanbyoul Cho, Doo Byung Chay, Bang Hyun Lee, Seong Jin Cho, Soonwon Hong and Jae-Hoon Kim

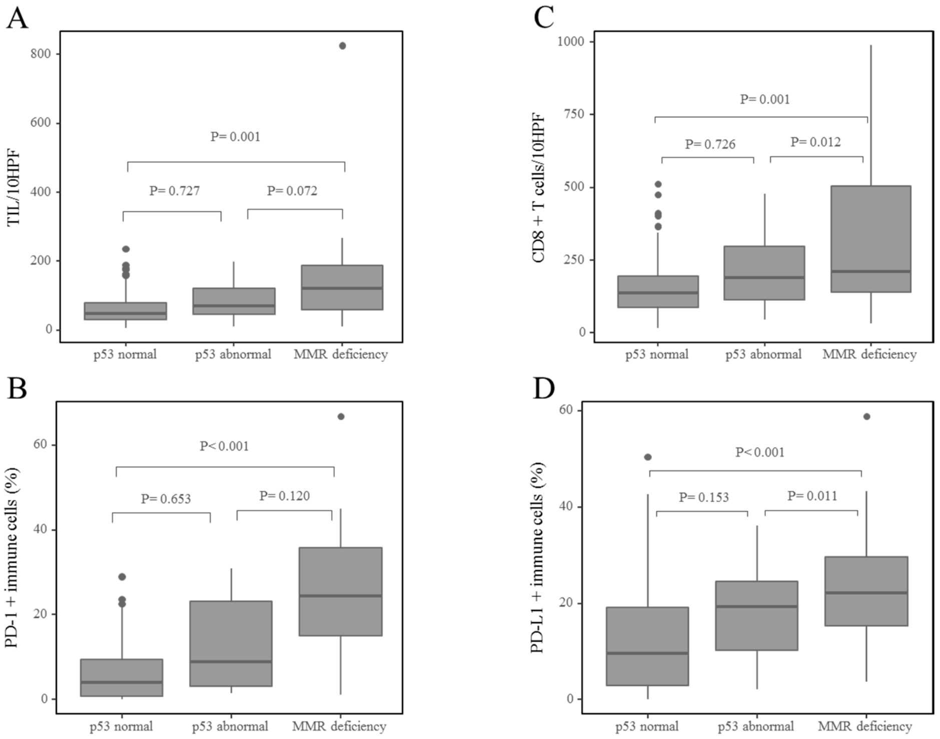

Figure S1. Peritumoral immune marker expression according to the practical molecular classification of endometrial cancer based on DNA mismatch repair (MMR) status and p53 mutation status in endometrial cancer patients. (**A**) TIL, (**B**) $CD8^+$ T lymphocytes, (**C**) peritumoral $PD\text{-}1^+$ immune cells, and (**D**) peritumoral $PD\text{-}L1^+$ immune cells.

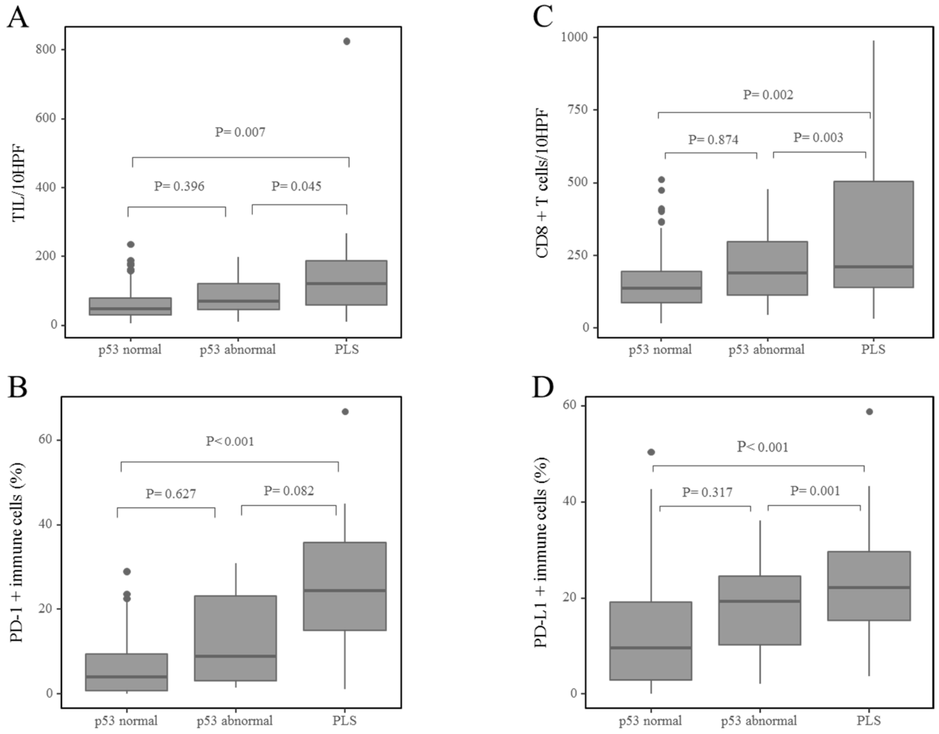

Figure S2. Peritumoral immune marker expression according to the practical molecular classification of endometrial cancer based on probable Lynch syndrome (PLS) status and p53 mutation status in endometrial cancer patients. (**A**) TIL, (**B**) CD8^{+} T lymphocytes, (**C**) peritumoral PD-1^{+} immune cells, and (**D**) peritumoral PD-L1^{+} immune cells.

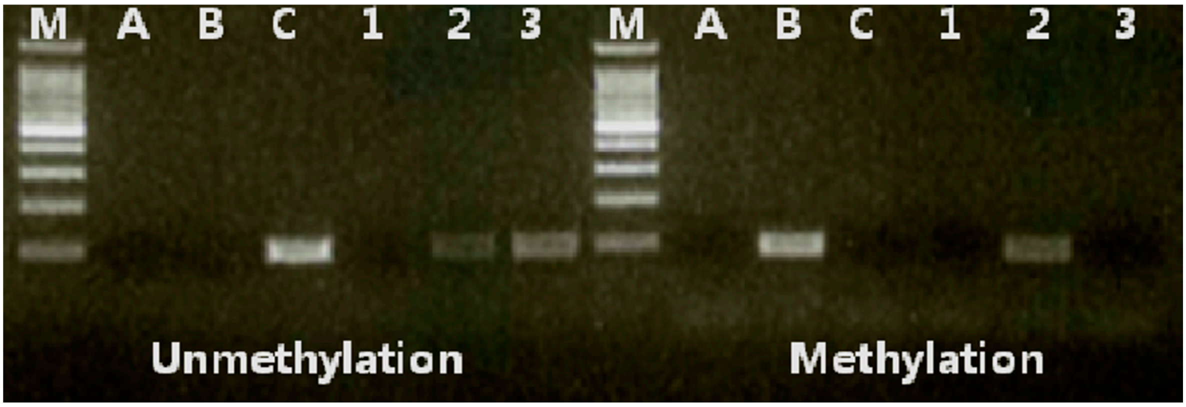

Figure S3. Results of MLH1 promotor methylation test showing amplified un methylation and methylation PCR products. A: Unmethylated human control DNA MLH1 methylation PCR; B: Methylated human control DNA (bisulfite converted); C: Unmethylated human control DNA (bisulfite converted); 1: Not significant result due to material insufficiency; 2: A case showing MLH1 methylation; 3: A case showing no MLH1 methylation.

Table S1. European Society of Medical Oncologists (ESMO) risk groups [1]

Risk group	Description
Low	Stage I endometrioid, grade 1–2, <50% myometrial invasion, LVSI negative
Intermediate	Stage I endometrioid, grade 1–2, ≥50% myometrial invasion, LVSI negative
High-intermediate	Stage I endometrioid, grade 3, <50% myometrial invasion, regardless of LVSI status
	Stage I endometrioid, grade 1–2, LVSI unequivocally positive, regardless of depth of invasion
High	Stage I endometrioid, grade 3, ≥50% myometrial invasion, regardless of LVSI status
	Stage II
	Stage III endometrioid, no residual disease
	Non-endometrioid (serous or clear-cell or undifferentiated carcinoma, or carcinosarcoma)
Advanced	Stage III residual disease and stage IVA
Metastatic	Stage IVB

FIGO 2009 staging used; LVSI, lymphovascular space invasion.

References

1. Colombo, N.; Creutzberg, C.; Amant, F.; Bosse, T.; Gonzalez-Martin, A.; Ledermann, J.; Marth, C.; Nout, R.; Querleu, D.; Mirza, M.R.; et al. Esmo-esgo-estro consensus conference on endometrial cancer: Diagnosis, treatment and follow-up. *Ann. Oncol.* **2016**, *27*, 16–41.
